# Idioventricular Rhythm: A Rare Presentation of Left Ventricular Pseudoaneurysm Following Radiofrequency Ablation

**DOI:** 10.7759/cureus.55272

**Published:** 2024-02-29

**Authors:** Bright E Izekor, Jessica Lovelace, Thao K Giang, Elizabeth Ebert, Gregory Olsovsky

**Affiliations:** 1 Cardiology, Baylor Scott & White Medical Center - Temple, Temple, USA; 2 Internal Medicine, Baylor Scott & White Medical Center - Temple, Temple, USA; 3 Electrophysiology, Baylor Scott & White Medical Center - Temple, Temple, USA

**Keywords:** ventricular arrhythmias, idioventricular rhythm, premature ventricular complexes, radiofrequency ablation (rfa), left ventricular pseudoaneurysm

## Abstract

Left ventricular pseudoaneurysm (PsA) is a rare complication of radiofrequency ablation (RFA) of cardiac arrhythmias. Presentation can vary widely in terms of timeline, signs, and symptoms. Idioventricular rhythm is a rare presentation of PsA post-ablation. No cases of post-ablation PsA presenting with idioventricular rhythm have been reported in the literature to date.

A 72-year-old male presented with symptomatic idioventricular rhythm 34 days post RFA for premature ventricular complexes (PVCs). A PsA involving the distal anterolateral of his left ventricle wall was identified on transthoracic echo and computed tomography (CT). This patient underwent surgical patch repair which resolved his ventricular arrhythmia.

## Introduction

Left ventricular pseudoaneurysms (PsAs) are an uncommon but life-threatening cardiac pathology. They are most associated with myocardial infarction, cardiac surgery, trauma, and endocarditis [1]. A systematic review by Inayat et al. reported myocardial infarction to be most associated with PsA, accounting for up to 55% of all cases [2]. Unlike a true aneurysm, PsA of the left ventricle contains no endocardium or myocardium and is believed to form because of wall stress, loss of myocardial integrity, or contained myocardial rupture [3]. Several imaging modalities can aid in diagnosing left ventricular PsA. These include angiography, transthoracic echocardiography, transesophageal echocardiography, cardiac MRI, and cardiac computed tomography (CT) [1]. Cardiac angiography provides the most definitive diagnosis, with Frances et al. reporting a specificity greater than 85% [1]. Complications of untreated left ventricular PsA include arrhythmias, thromboembolisms, congestive heart failure, tamponade, shock, and death [1,3].

Radiofrequency ablation (RFA) is a very rare cause of left ventricular PsA. Only eight cases of PsA occurring following RFA have been reported to date [4-11]. The presentation can vary considerably. While ventricular arrhythmias have been associated with PsAs from other etiologies, there are currently no reports in the literature of PsAs post-RFA presenting with ventricular arrhythmia.

## Case presentation

**Figure 1:**
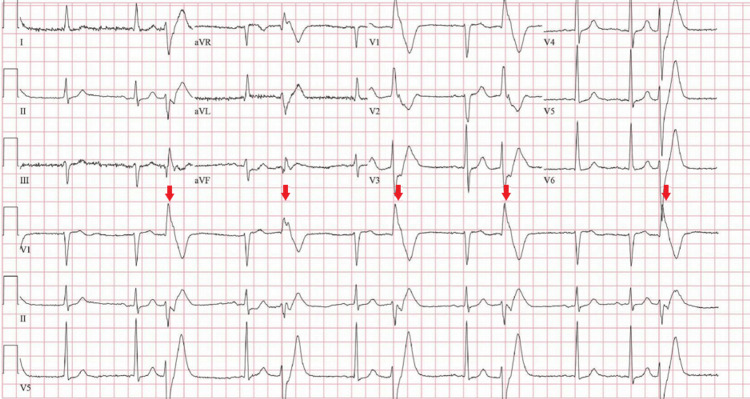
Initial ECG showing frequent PVCs (red arrows) with RBBB morphology and superior axis. PVCs: premature ventricular complexes, RBBB: right bundle branch block

Our patient is a 72-year-old male with a history of atrial fibrillation who initially presented to his primary electrophysiologist with complaints of palpitations. An electrocardiogram (ECG) done at that time showed premature ventricular complexes (PVCs) in a bigeminy pattern; there was right bundle branch block (RBBB) morphology, superior axis, and transition in lead V3 (Figure 1).

The initial plan was to continue medical therapy with diltiazem and flecainide with a reassessment in six months. However, he called back nine days later with complaints of persistent palpitations, dyspnea, and fatigue. ECG showed PVCs with similar morphology and axis in a trigeminy pattern. His chest x-ray did not show any acute cardiopulmonary abnormalities; his echocardiogram showed mildly reduced ejection fraction at 47% without regional wall motion abnormalities; his other workup, including troponin and brain natriuretic peptic peptide (BNP), was within normal limits. He was admitted for monitoring. During this admission, he was evaluated by our inpatient electrophysiology team, and a shared decision was made to pursue electrophysiologic study and RFA. 

**Figure 2:**
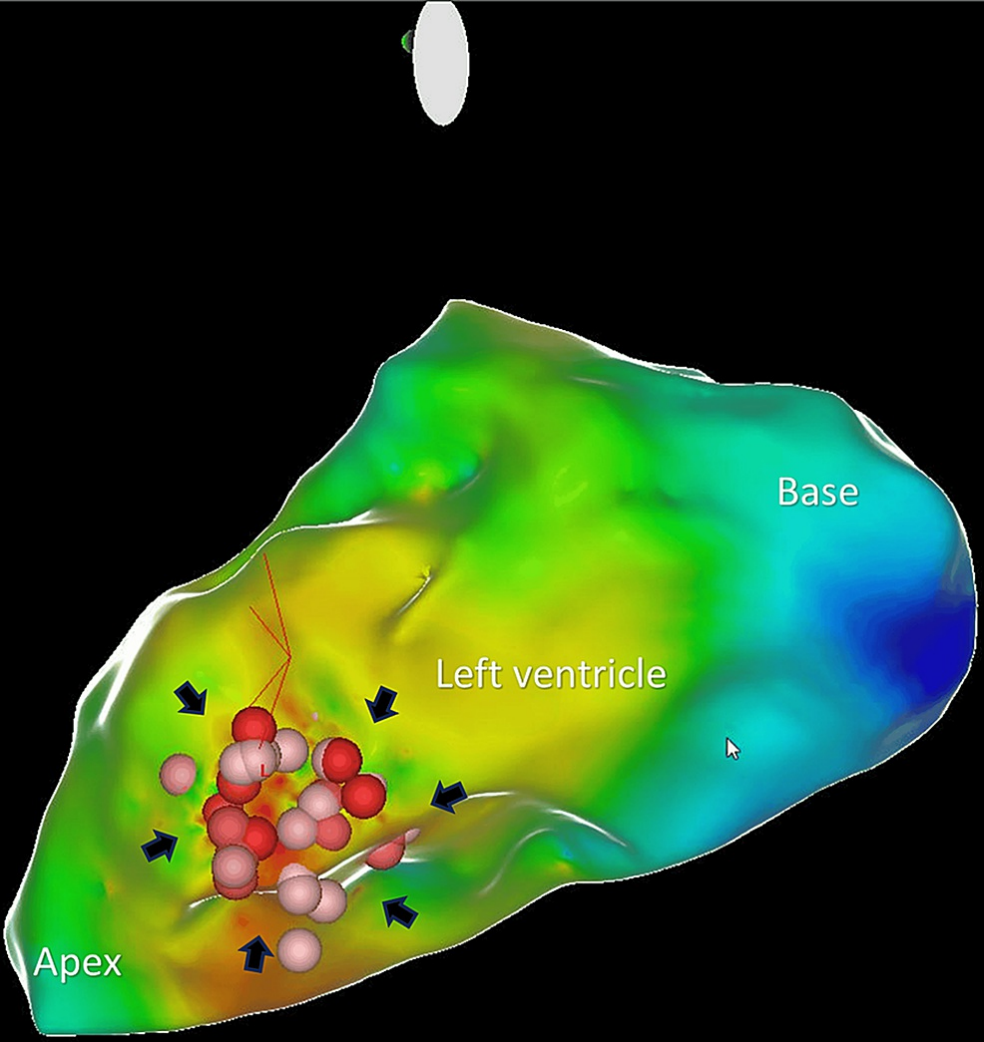
Activation timing map of left ventricle showing area of radiofrequency ablation along the distal anterolateral wall (black arrows).

An electrophysiologic study was performed with baseline intervals recorded. Reconstruction of the left ventricle was done using the Biosense Webster Carto system. The activation timing map revealed the earliest activation to be near the posterior medial papillary muscle. Using a Thermocool RMT catheter (Biosense Webster), radiofrequency energy was applied at the location of the earliest location at a power of 40 Watts for 20-60 seconds in duration (Figure 2).

Unfortunately, the PVCs persisted despite extensive ablation in that region and utilization of higher energy, up to 50 Watts. There was an ablation application that resulted in a sharp increase in impedance for about one second; at this point, ablation applications were immediately discontinued. In total, 30 ablation applications were done. There was no audible steam pop or evidence of pericardial effusion after ablation applications. He was observed overnight and discharged with increased medical therapy.

**Figure 3:**
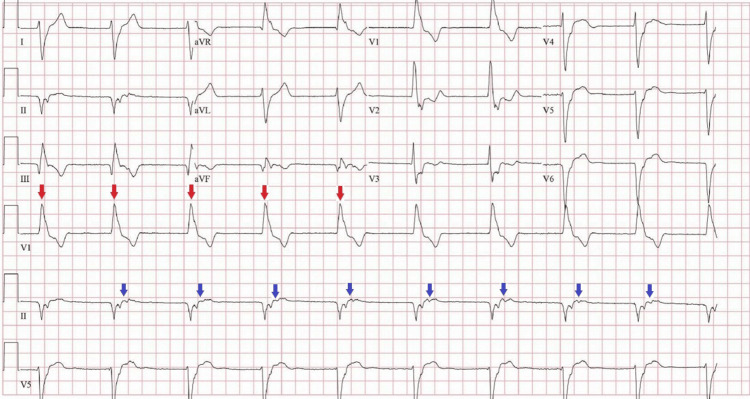
ECG on admission showing wide-complex rhythm with retrograde p waves (blue arrows) consistent with idioventricular rhythm. There is also a right bundle branch block pattern (red arrows indicating upright QRS in V1) and a superior axis suggesting that this rhythm was likely originating from the same location as his PVCs.

He had a follow-up appointment four days post-discharge where he reported doing well; an ECG done during this visit showed the persistence of PVCs. He presented to the ED with palpitations 34 days later. ECG done during this encounter showed an accelerated idioventricular rhythm (Figure 3).

**Figure 4:**
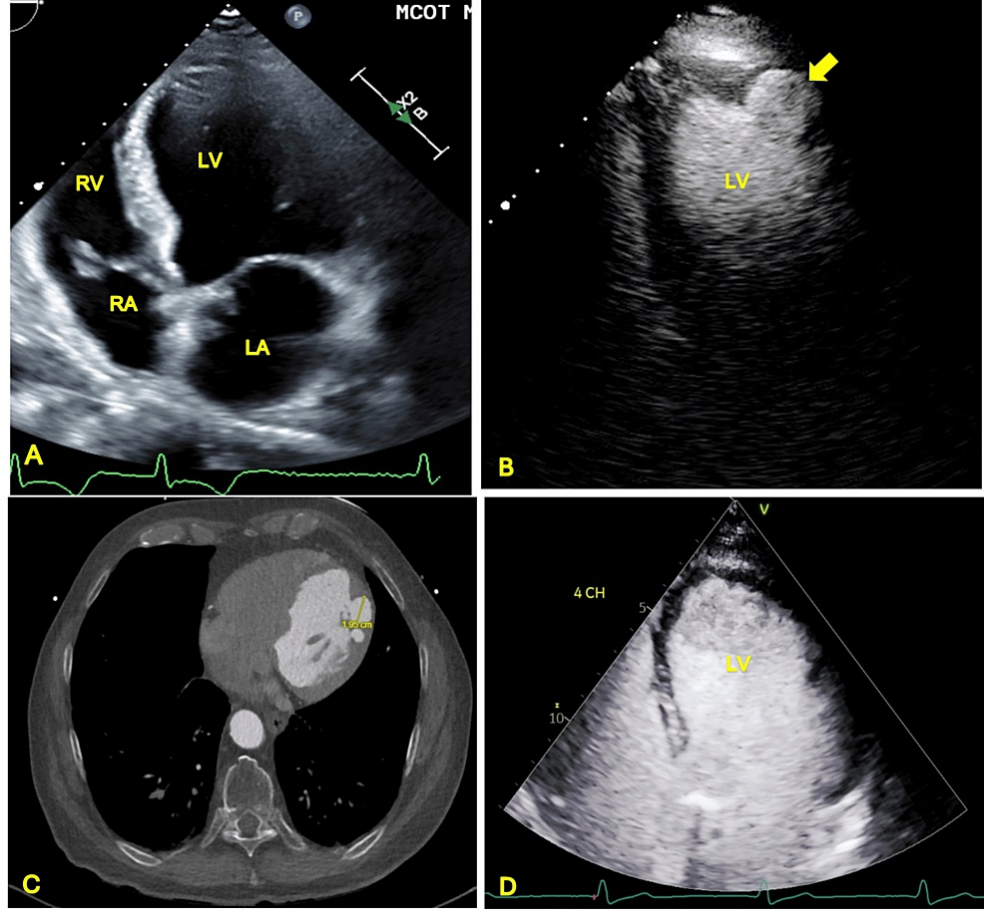
(A) Apical four-chamber view of transthoracic echocardiogram without contrast enhancement. Pseudoaneurysm was not apparent in this view. (B) Apical four-chamber view with contrast enhancement demonstrating left ventricular pseudoaneurysm (yellow arrow) in the distal anterolateral wall. (C) Contrast-enhanced CT demonstrating pseudoaneurysm in the distal anterolateral wall of the left ventricle measuring 1.95 cm at its largest diameter. (D) Apical four-chamber view of transthoracic echocardiogram with contrast enhancement post pseudoaneurysm repair. This repeat image demonstrates that pseudoaneurysm has resolved with surgical repair. LV: left ventricle, LA: left atrium, RV: right ventricle, RA: right atrium

An echocardiogram was obtained which showed a left ventricular PsA, measuring 2 cm in AP diameter, on the anterolateral wall; the PsA was only apparent after contrast enhancement (Figures 4A, 4B).

A contrast-enhanced CT was obtained which also demonstrated a PsA at the same location (Figure 4C). There were concerns about myocardial ischemia being the etiology of his presenting rhythm and echo findings. This prompted noninvasive cardiac evaluation with a myocardial perfusion scan which showed a fixed defect in the distal anterolateral wall consistent with a myocardial scar. He then underwent a coronary angiogram which showed mild single-vessel coronary artery disease (CAD) involving the mid-left anterior descending artery; there was no angiographic evidence of CAD in the left circumflex and right coronary arteries. At this point, it was deemed that his PsA was likely related to his recent PVC ablation.

Cardiothoracic surgery was consulted. He was taken to the operating room where a large left ventricular PsA was seen intraoperatively with thrombus present. A patch repair was done without complications. He was initially atrial-paced post-operatively with ECG prior to discharge showing only occasional PVCs; no further episodes of idioventricular rhythm were observed during this admission. A follow-up echo showed that the PsA had resolved (Figure 4D). He was discharged on post-op day 9 with a mobile cardiac telemetry unit (MCOT). No evidence of ventricular arrhythmia was present on MCOT at the 30-day follow-up.

## Discussion

While left ventricular PsAs are generally known to be rare, even rarer are left ventricular PsA following RFA. From our review, only eight cases have been reported in the literature [4-11]. The earliest case was published in 2001 by Gill et al. which involved an incidental small left ventricular PsA in an asymptomatic patient post-RFA ablation of an accessory pathway [4]. The following published case was by Mansour et al., in 2006, of a symptomatic patient who presented with a sizeable left ventricular PsA 15 years post ablation of an accessory pathway [5]. Mansour et al.’s case showed the widely variable timeline of presentation with left ventricular PsA. Unlike the case published by Gill et al. where the PsA spontaneously resolved, the case presented by Mansour et al. required surgical resection, as did all but two subsequent reported cases. Left ventricular PsA complicating RFA ablation of ventricular arrhythmia was recognized relatively recently. All six published cases have been within the last ten years. The first published case was by Koch et al. in 2015. This case involved a patient who developed a 6.1 cm left ventricular PsA following multiple RFAs of ventricular tachycardia. CT angiogram of the heart was used to characterize the PsA after initial diagnosis with a transthoracic echocardiogram. Koch et al. highlight cardiac CT's role in diagnosing and managing left ventricular PsA [6].

In our case, our patient developed PsA over a month post-PVC ablation. Interestingly, the initial presentation was idioventricular rhythm. In two of the eight previously reported cases, PsA diagnosis was incidental [4,7]. In three cases, the presenting symptom was chest pain [8-10]. In the remaining cases, presentations included syncope [5], atrial fibrillation [6], and cardiac tamponade [11]. So far, there have been no reports of post-RFA PsA presenting as ventricular arrhythmia, making our case unique. We concluded that the presenting ventricular arrhythmia was likely scar-mediated; this was supported by myocardial perfusion scan findings of a fixed defect in an area that corresponds with the location of the left ventricular PsA. In this case, we deemed that scar formation was likely related to extensive RFA at that site. We also believe that there may have been an inaudible steam pop (evidenced by the sudden increase in impedance) which led to myocardial damage predisposing to PsA formation. As such, there should be a high index of suspicion for left ventricular PsA in all patients presenting with ventricular arrhythmia following RFA, especially in cases that require repeated and/or extensive ablation and concerns for steam pop. Also interesting about this case is that the PsA was not readily visible on 2D echocardiography until contrast (definity) was administered. This underscores a possible role for routine use of contrast enhancement on all follow-up echocardiography post-RFA. Our patient was ultimately managed with surgical patch repair. The follow-up transthoracic echocardiogram did not show any evidence of residual PsA. He was discharged with a mobile cardiac telemetry unit, which showed no recurrence of ventricular arrhythmia.

## Conclusions

Left ventricular PsA is a rare complication of RFA, and presentation can vary widely. To date, there are no reported cases of post-RFA left ventricular PsA presenting with idioventricular rhythm. Thus, it is important to have a high clinical suspicion for left ventricular PsA in patients presenting with ventricular arrhythmias following RFA. In addition, routine use of contrast enhancement in follow-up echocardiography for post-RFA patients may help further rule out this life-threatening complication. 
